# Brugada syndrome update

**DOI:** 10.3389/fphys.2024.1520008

**Published:** 2025-01-17

**Authors:** Tingting Xu, Shaokun Wang, Jiawen Wang, Jihong Xing

**Affiliations:** ^1^ Department of Emergency Medicine, The First Hospital of Jilin University, Changchun, Jilin, China; ^2^ Department of Nuclear Medicine, The First Hospital of Jilin University, Changchun, Jilin, China

**Keywords:** Brugada syndrome, right ventricular outflow tract, quinidine, implantable cardioverter defibrillator, radiofrequency ablation

## Abstract

Brugada syndrome (BrS), a genetic disorder affecting cardiac ion channels, predominantly manifests due to mutations that impair the function of the Nav1.5 sodium channel’s α-subunit. This condition, identified by Josep and Pedro Brugada, is often marked by symptoms such as syncope and episodes of polymorphic ventricular tachycardia (PVT) or ventricular fibrillation (VF). These arrhythmias, if not managed promptly, can escalate to sudden cardiac death (SCD), notably in patients whose cardiac structure appears normal. Given this, the prompt recognition and stratification of individuals at elevated risk are critical. This review elaborates on the current insights into BrS, focusing on recent diagnostic techniques, risk assessment strategies, and therapeutic advancements. It also critically examines ongoing controversies in the field.

## 1 Introduction

BrS, first described by Josep and Pedro Brugada in 1992, is a genetic disorder affecting cardiac function, predominantly inherited in an autosomal dominant manner (Cerrone et al., 2022). The initial discovery involved a group of eight individuals who survived SCD events attributed to VF (Adler, 2016). This condition was identified by the characteristic presence of persistent ST segment elevation combined with right bundle branch block, which was later recognized as indicative of a predisposition to SCD. The syndrome was formally named Brugada syndrome in 1996 following these findings (Brugada et al., 2018). BrS has been associated with 4%–12% of all SCD occurrences and is responsible for approximately one-fifth of SCD cases in patients with structurally normal hearts (Juang and Huang, 2004). These statistics emphasize the importance of BrS in understanding the mechanisms underlying cardiac arrhythmias and improving strategies for diagnosis and clinical management in cardiology. The overall global prevalence of BrS is 0.5/1,000 (Vutthikraivit et al., 2018), with the highest prevalence 3.7/1,000 in Southeast Asia and 17.7/1,000 in Thailand (Vutthikraivit et al., 2018; Rattanawong et al., 2020). The clinical manifestations are more commonly appeared in adults at the age of 40 years and over. And the incidence in men is 8–10 times that in women (Brugada et al., 2018; Pappone and Brugada, 2017), which maybe related to male testosterone concentration (Matsuo et al., 2003). The following overview details the pathophysiology, genetics, clinical manifestations, predisposing factors, diagnosis, differential diagnosis, risk stratification, treatment and updates on BrS in specific populations.

## 2 Genetics

BrS is acknowledged as a hereditary cardiac condition, with familial SCD documented in approximately 26% of individuals diagnosed with the syndrome (Polovina et al., 2017). Genetic analysis reveals that BrS transmits via an autosomal dominant pathway, albeit with incomplete penetrance (Cerrone et al., 2022). Typically emerging in adulthood, particularly in the third or fourth decades, the condition can manifest at any stage from infancy through to late adulthood (Brugada et al., 2018; Mazzanti and Priori, 2016). In a landmark study conducted in 1998, Chen and colleagues pinpointed a critical genetic mutation within the SCN5A gene. This gene is responsible for producing the α subunit of the NaV1.5, the cardiac voltage-gated sodium channel, which is crucial for the initiation of phase 0 in the cardiac action potential (Chen et al., 1998). This gene’s mutations, including frameshifts and disruptions that prevent channel expression, lead to significantly reduced sodium current (INa) density (Kabra et al., 2020). Recent findings by Ciconte G et al. have established a correlation between pathogenic variants in the SCN5A gene and the severity of arrhythmogenic substrates in the epicardial aspect of the right ventricular outflow tract (RVOT). Individuals carrying these variants exhibit pronounced epicardial electrical anomalies and present a more severe clinical course (Ciconte et al., 2021). Initially regarded as a simple autosomal dominant monogenic condition necessitating just one mutational event, BrS is currently viewed as potentially oligogenic or polygenic, with multiple genetic modifiers influencing the severity and manifestation of the primary genetic anomaly (Cerrone et al., 2019). The expansion of gene detection methodologies, along with the advent of high-throughput next-generation sequencing, has led to an exponential increase in the volume of genetic data available (Isbister et al., 2020). To date, researchers have identified over 500 pathogenic variants associated with BrS, with around 30% of these variants located within the SCN5A gene (Brugada et al., 2018). Approximately 5% of BrS diagnoses are attributable to mutations in a subset of genes affecting Na⁺, Ca^2^⁺, and K⁺ ion channels on cardiac cell membranes (Brugada et al., 2014). These mutations alter the currents of inward Na⁺ and Ca^2^⁺ ions, and outward K⁺ ions (Korlipara et al., 2021). Such disruptions may lead to a loss of action potential in the right ventricular epicardium, potentially triggering ventricular arrhythmias (Kabra et al., 2020). The gene mutations that regulate sodium channel current were found to be in *SCN5A*, *SCN10A*, *HEY2*, *PKP2*, *GPD1-L*, *RANGRF*, *SLMAP*, *SCN1B*, *SCN2B*, *SCN3B*, *SCN4A* and *KCNE3* (Korlipara et al., 2021); The gene mutations that regulate calcium channel current were found to be in *CACNA1C*, *CACNB2B*, *CACNA2D1* and *TRPM4* (Korlipara et al., 2021); The gene mutations that regulate potassium channel current were found to be in *KCNE3*, *KCNJ8*, *KCND3*, *KCNE5*, *ABCC9*, *HCN4* and *SCN1B* (Korlipara et al., 2021). A comprehensive overview of the genetic insights associated with BrS is provided in Table 1.

**TABLE 1:** Genetics of Brugada syndrome.

BrS susceptibility genes	Prevalence in BrS cases	The main role of mutant genes
*SCN5A*	30%	*SCN5A* was the first gene found to be associated with BrS. Mutations encodes the α-subunit of the cardiac sodium channel, result in a decrease in NaV1.5 α-subunit protein levels, impaired sodium channel functionality, and weakened phase 0 action potentials
*SCN10A*	About 5%	This gene encodes the neuronal Na^+^ channel (NaV1.8) expressed in the myocardium, It also leads to a significant reduction in INa
*HEY2*		In the cardiovascular system, *HEY2* encodes a helix-loop-helix transcriptional repressor.*HEY2* deficiency affects the permeabilised expression gradient of BrS-associated NaV1.5 channels
*PKP2*		*PKP2* stands as the primary gene linked to arrhythmogenic right ventricular cardiomyopathy, and its absence diminishes sodium channel activity, impacting myocyte interactions
*GPD1-L*		The gene is responsible for producing a protein similar to glycerol-2 phosphate dehydrogenase-1.Fluctuations in GPD1-L lead to decreased expression on the surface membrane and lower inward sodium flows in NaV1.5
*RANGRF*		Alterations in the gene responsible for producing MOG1, a protein controlling NaV1.5, disrupt the movement of NaV1.5 to the cellular membrane, leading to decreased sodium flow
*SLMAP*		Pathogenic variants of this gene are present in T-tubules and sarcoplasmic reticulum and cause BrS by regulating intracellular transport of NaV1.5 channels
*SCN1B*		The gene is responsible for producing the β1 component of the NaV1.5 sodiumion channel. Alterations in this gene may lead to a concurrent reduction in INa and a rise in ITo
*SCN2B*		This gene encodes the β2 subunit of the NaV1.5 channel, and mutations in this gene result in a significant reduction in sodium current density
*SCN3B*		This gene encodes the β3 subunit of the cardiac NaV1.5 channel. Mutations in this gene result in reduced sodium current density, impairing cell surface expression and function of sodium channels
*SCN4A*		The SCN4A gene is responsible for producing the alpha subunit of the NaV1.4 voltage-gated sodium channel in skeletal muscles, and alterations in this gene are linked to muscular sodium channel disorders
*CACNA1C*		This gene encodes the alpha subunit of the L-type voltage-gated calcium channel CaV1.2, and altering it leads to the cessation of calcium channel activity
*CACNB2B*		Mutations in this gene, which encodes the β2 subunit of the type L voltage-gated calcium channel CaV1.2, accelerate the deactivation of L-type calcium currents and diminish the maximum calcium current
*CACNA2D1*		This gene encodes the α2β subunit of voltage-gated calcium channels, plays a crucial role in controlling current density and the activation or deactivation of these channels. Mutations in this gene have been shown to be associated with BrS
*TRPM4*		This gene encodes a calcium-activated transient receptor potential. Both gain and loss of function of TRPM4 channels can reduce sodium channel activity and result in slowed conduction
*KCNE3*		The gene in question is responsible for producing the MinK-related peptide 2 (MiRP2) protein, which plays a key role in regulating the temporary external potassium current ITo
*KCNJ8*		This gene is responsible for encoding the Kir6.1 subunit, and its mutation results in enhanced epicardial KATP channel activity, reduced plateau phase and shorter action potential duration
*KCND3*		The gene is responsible for producing the α-subunit of the ITo channel, and a rise in functional mutations within this gene further amplifies the dynamics of ITo
*KCNE5*		This gene is responsible for producing a regulatory β-subunit in the Ito and IKs channels. Alterations in this condition are linked to arrhythmias induced by BrS
*ABCC9*		The gene is responsible for producing SUR2A, which is the transporter for the ATP-binding cassette in the IK-ATP channel. Mutations in this gene reduce KATP sensitivity
*HCN4*		Mutations in the hyperpolarisation-activated cyclic nucleotides of potassium channels, produced by the HCN4 gene, are linked to BrS, potentially causing sinus node malfunction and diminished pacemaker accessibility
*SCN1B*		Alterations in the gene responsible for the β1 component of the NaV1.5 sodium channel lead to increased temporary potassium flows outward and diminished maximum sodium currents

References: (Cerrone et al., 2022; Brugada et al., 2018; Polovina et al., 2017; Mazzanti and Priori, 2016; Brugada et al., 2014; Korlipara et al., 2021; Antzelevitch et al., 2016a; Sotoodehnia et al., 2010; Hu et al., 2014a; Abriel, 2015; Nakano et al., 2016; Sarquella-Brugada et al., 2016; Campuzano et al., 2016; Olesen et al., 2011; Ishikawa et al., 2012; Probst and Gourraud, 2015; Olesen et al., 2012; Bao et al., 2016; Kurakami and Ishii, 2013; Bissay et al., 2016; Cordeiro et al., 2009; Liu et al., 2013; Giudicessi et al., 2011; Medeiros-Domingo et al., 2010; Hu et al., 2014b; Ueda et al., 2009).

## 3 Pathophysiology

So, generation of the cardiac action potential (AP) is a complex interaction of various ion channels, the ionic milieu surrounding, the membrane potentials and the regulatory proteins (Moras et al., 2023). Fast sodium channels generate a large and fast INa that, along with transient Cav1.2 channels, result in membrane depolarisation during the initial upstroke of the AP (Moras et al., 2023). Cardiac action potential repolarization is composed of complex, sequential events that are orchestrated via a precisely tuned balance between the inward depolarizing currents of ion channels and outward repolarizing currents. The dominant depolarizing currents during the extended plateau phase of the AP are mainly facilitated by ICaL. Due to increasing enhancement of the IKr and IKs currents that peak during the latter part of the plateau, the balance of channel currents progressively shifts toward more outward repolarization (Moras et al., 2023). Alterations in ion channels that are important in generation of cardiac action potentials are recognised as an underlying cause of BrS, thus leading to the channelopathy categorization of this disease. It includes disruption of the normal function of INa, ICaL, or transient outward potassium channels (ITo) (Cerrone et al., 2022). A dysfunction to these currents (attenuation or augmentation) causes a predominant change on the temporospatial dominance in the activation of outward currents at the beginning of the action potential of the RVOT (Cerrone et al., 2022).

To date, the understanding of BrS′s underlying cellular mechanisms and pathophysiology has been shaped by three leading hypotheses: The syndrome has been attributed to abnormal repolarization processes within cardiac cells (the Repolarization Hypothesis), to abnormalities of depolarization (the Depolarization Hypothesis), and also by problems with development of neural crest cells (the Neural Crest Hypothesis17) (Korlipara et al., 2021).

### 3.1 The Repolarization hypothesis

It is generally believed that the electrophysiological mechanism of BrS is based on regional differences in the electrophysiological properties of myocardial cells (Isbister et al., 2020). Therepolarization hypothesis states that its electrophysiological mechanism is primary repolarization disorder caused by abnormally shortened duration of epicardial AP (Di Diego et al., 1996). A decrease in the sodium current can result in a relative augmentation of ITo, which subsequently repolarizes the membrane past the threshold where ICaL activates, thus precipitating the disappearance of the AP dome (Vlachos et al., 2020). Moreover, a notable transmural gradient from the epicardium to the endocardium has been documented, which tends to manifest as characteristic saddle-shaped or arch-like elevations on the ST-segment, and these are often paired with positive T-waves (Kabra et al., 2020). This gradient was first suggested by Yan and Antzelevitch in 1999 as a critical factor in facilitating cardiac re-entry through what is known as a phase 2 re-entrant pathway (Popa et al., 2023). The mechanism of phase 2 re-entry includes electrotonic interactions that facilitate the propagation of the action potential from regions of the epicardium that exhibit a pronounced AP dome to adjacent regions where this dome is absent (Maoz et al., 2014). This phenomenon of AP dome extension beyond the boundaries of the epicardial to the endocardial regions is thought to explain the mechanisms behind the observed ST-segment elevation and the inversion of T-waves (Meregalli et al., 2005; Coronel et al., 2005).

### 3.2 The depolarisation hypothesis

The prevailing hypothesis regarding the electrical disturbances in BrS centers on the reduced inward depolarization current, potentially exacerbated by structural anomalies located primarily beneath the pericardium of the RVOT (Wilde et al., 2010). Before the action potential is triggered in the RVOT, the right ventricle, already in a state of depolarization, serves as a current source, directing flow towards the RVOT and resulting in a positive deflection on the ECG (Nagase et al., 2002). Substantial clinical evidence supports this theory, illustrating that BrS frequently displays various signs of slowed electrical conduction on ECG tracings (Cerrone et al., 2022). The research focus has recently broadened to include detailed analyses of late potentials, which are markedly prevalent among patients with BrS (Cerrone et al., 2022). Key evidence supporting the depolarization hypothesis includes studies by Nademanee et al. (2011), which reported the detection of late potentials and fractionated electrograms in the RVOT of BrS patients through bipolar electrograms. These findings imply that the patterns in unipolar recordings at the RVOT’s anterior epicardial wall stem from areas of significantly reduced conduction velocity (Cerrone et al., 2022). In another pivotal prospective study with 250 BrS patients, Ciconte et al. (2019) established a correlation between epicardial abnormal substrate (AS) and the identification of late potentials in signal-averaged electrocardiography (SAECG), highlighting these potentials as markers of abnormal epicardial electrophysiological behavior. The study reported impressive diagnostic performance, evidenced by an area under the curve of 0.88, sensitivity of 86%, specificity of 88%, a positive predictive value of 85%, and a negative predictive value of 89%. The high negative predictive value notably indicates the utility of this approach in identifying individuals with less pronounced arrhythmic substrates. Of note, late potentials are more commonly observed in symptomatic BrS patients, and SAECG may be considered part of a diagnostic test for risk stratification of BrS patients.

### 3.3 The neural crest hypothesis

Elizari et al. (2007) introduced the neural crest hypothesis, indicating that cells derived from the neural crest reside in regions beyond the cardiac area. These cells play a pivotal role in the development of the myocardium within the RVOT and in forming its surrounding structures. BrS may be associated with improper expression of neural crest cells and their neighboring tissues during the embryonic development of the RVOT. It is suggested that aberrant expression of cardiac neural crest cells could result in altered connexin expression, particularly Cx43, leading to conduction and activation delays in the RVOT observed in BrS.

Although standard cardiac echocardiography frequently indicates normal heart anatomy in patients, the utilization of more advanced imaging modalities such as MRI and CT has led to the detection of subtle structural anomalies in individuals diagnosed with BrS. These intricate irregularities encompass the enlargement of the RVOT, a reduced ejection fraction of the RV), and disturbances in the motion of the RV wall (Marsman et al., 2022). Furthermore, a ECGs from BrS patients has revealed structural abnormalities within the RV, as evidenced by the study conducted by Catalano et al. (2009). In their research, Catalano and colleagues performed a comparative analysis of MRI scans from a cohort of 30 BrS patients against those from a control group of healthy individuals matched for relevant demographic and clinical characteristics. The findings demonstrated that the BrS group exhibited a markedly higher prevalence of mild structural modifications in the RV, suggesting a potential association between BrS and these subtle cardiac structural changes. Pieroni et al. (2018) reported fibrosis in 15 RVOT specimens from 20 BrS patients. Miles et al. (2021) found an increased proportion of collagen in RVOT, up to 24%.

The diminished conduction capacity of the RVOT has been suggested as a converging endpoint for all previously discussed mechanisms (Marsman et al., 2022). Factors influencing this conduction capacity include demographic variables such as age, sex, and ethnicity, as well as the existence of structural anomalies in tissues and/or dysfunctions in ion channels. Additionally, this conduction capacity is modulated by a number of external modulators like body temperature, pharmacological agents and vagal tone changes (Marsman et al., 2022; Behr et al., 2021; Blok and Boukens, 2020), and the collective effect of these modulators also contributes to the BrS phenotype. Therefore, in the end, the interaction of a multitude of electrophysiological characteristics, structural anomaly, genetic preposition, and the environment together determine both the expression and the strength of the BrS phenotype (Marsman et al., 2022).

An important investigation (Tarantino et al., 2024) by Tarantino A et al., consisted on the screening of 50 patients BrS (Tarantino et al., 2024). They discovered that 90% of these BrS patients had NaV1.5 autoantibodis, in stark contrast to 6% among the control cohort. Moreover, detection of anti-NaV1.5 antibody IgG was not correlated with a SCN5A gene mutation, or with age, sex, or specific electrocardiogram (ECG) patterns of patients. The analysis was also expanded in the research to plasma samples from an additional 35 patients with different cardiac conditions such as long QT syndrome, structural cardiomyopathy and heart failure. No NaV1.5 autoantibodies were found in this group. The study diagnostic performance was notably robust with high specificity (94%) and sensitivity (90%), an area under the curve (AUC) of 0.92, and 100% PPV and 97.5% NPV. In addition, animal studies which injected plasma from BrS patients into mice showed that these mice developed Brugada‐like ECG abnormalities. Following this, we reconsidered the pathological mechanism of BrS and the detection of NaV1.5 autoantibodies improved diagnostic accuracy and introduced another path to BrS diagnosis. The opening of new avenues for the treatment of BrS by immunotherapy to inhibit NaV1.5 autoantibodies has also been introduced. Pluripotent stem cells (iPS-CM) derived cardiomyocytes have been intensively studied as an innovative experimental approach in recent years. Yamanaka et al. (Park et al., 2008; Takahashi and Yamanaka, 2006) have pioneered work demonstrating the ability to revert somatic cells to a state of pluripotency by introducing multiple transcription factors (e.g., Oct4, Sox2 and Myc) required for pluripotency and cell proliferation. “Ideally, this breakthrough opens the way for making cells with specific genetic properties from the patient’s own sample.” And then, these reprogrammed iPS cells can be induced to differentiate into particular cell types involved in different diseases, for example, differentiating to cardiomyocytes in the context of heart related conditions (Sendfeld et al., 2019). This methodology holds significant promise for enhancing our understanding of disease mechanism and can conceivably enrich future work into the pathogenesis of a wide variety of conditions with a robust, cell specific model of the disease.

## 4 Clinical manifestations

Most BrS patients are asymptomatic but present a wide variety of clinical presentations (Brugada et al., 2018). Symptoms such as syncope, epilepsy, sensations of chest discomfort, and abnormal respiration during sleep arising from—invariably—if not PVT, then VF are commonly observed (Brugada et al., 2018). If arrhythmias persist, individuals affected by these arrhythmias are at risk for developing SCD (Brugada et al., 2018; Korlipara et al., 2021). In addition, supraventricular arrhythmias are seen in up to 20% of those with BrS, including atrial fibrillation, atrial flutter, atrioventricular node reentrant tachycardia, and the Wolf-Parkinson-White syndrome (Eckardt et al., 2001). Also, according to Sara et al. (D’Imperio et al., 2021a), research has shown that BrS is not only confined to the cardiac domain but might involve several systems of the body. This widespread expression of ion channels throughout so many tissues allows us to attribute the wider implications of this phenomenon, namely, thyroid disorders, various cancers, skeletal muscle sodium channel lesions, lamellar lesions, diabetes, and electrolyte disturbances as well as primary cardiac issues (Parisi et al., 2013; Sandorfi et al., 2013; Camacho Velásquez et al., 2017; Abdelghani et al., 2020).

## 5 Inducing factors

For BrS patients, many substances have proarrhythmic effects and need to be avoided. Certain antiarrhythmic medications are known to carry risks of inducing arrhythmias, and this includes most Class I agents with the exception of quinidine. Other drugs such as amiodarone, lidocaine, propranolol, and verapamil also exhibit proarrhythmic potential. Additionally, psychotropic medications like amitriptyline and lithium, as well as anesthetic agents including bupivacaine, procaine, and propofol, have been associated with these adverse effects. Therefore, it is important to give this kind of drugs with caution and follow up (Polovina et al., 2017). Besides pharmacological agents, sodium channel blockers such as ajmaline, flecainide, pilsicainide and propafenone can also trigger BrS. Other contributing factors include enhanced vagal tone, assorted metabolic disturbances, abnormalities of electrovlytes, periods of binge eating, and use of substances such as marijuana, alcohol, and cocaine. Furthermore, BrS manifestations have been identified as being triggered by febrile states (Tomé and Freitas, 2017; Postema et al., 2009).

## 6 Diagnosis

BrS is detected with ECG and ECG is the fundamental exploratory instrument for BrS. The diagnostic marker for BrS (Brugada et al., 2018) currently accepted is the presence of a Type 1 ECG pattern, spontaneously or after intravenous infusion of sodium channel blockers during pharmacological test. Alternatively, BrS diagnosis is confirmed by the administration of a sodium channel blocker that turns a Type 2 ECG pattern into a Type 1 pattern (Priori et al., 2013). In addition, Type 2 ECG findings are not specific enough to diagnose BrS, and further proof of the diagnosis is required (Coppola et al., 2021).

If one or more ST segment (≥2 mm) elevations occur in the V1 to V3 leads and present in the second, third, or fourth intercostal space along with negative T waves, then its ECG pattern is Type 1 (Li et al., 2020). Spontaneous or drug induced by the use of provocatives agents like sodium channel blockers, this distinctive ECG morphology can occur (Korlipara et al., 2021). The annual incidence of cardiac events among individuals exhibiting a Type 1 ECG varies based on their clinical presentation: Among those who have never experienced syncope, it is 0.5%, approximately 1.9% for those who have had syncope, 7.7% for patients who have had an aborted SCD and 0.5% for patients who remain asymptomatic (Antzelevitch et al., 2016a; Postema PGJEEp and arrhythmias, 2012).

(2) Commonly known as the “saddleback type,” the Type 2 ECG pattern is defined by an ST segment elevation of more than 2 mm, appearing in the right ventricular leads, particularly from V1 to V3, and it exhibits either positive or biphasic T wave forms (Brugada et al., 2018; Kabra et al., 2020).

It is crucial to recognize that ECG variations related to BrS can occur sporadically and may occasionally be obscured. Frequently, confirmation of a BrS diagnosis relies on obtaining several ECG recordings over an extended period (Cerrone et al., 2022). Moreover, performing ECGs from the second intercostal space has the potential to produce false-positive Brugada patterns.

In cases where BrS is clinically suspected based on symptoms such as syncope, agonal breathing, aborted SCD, a family history suggestive of BrS, or inconclusive ECG findings, and where no spontaneous Type 1 ECG pattern is apparent, it is advisable to conduct a pharmacological test using a sodium channel blocker. The availability of these agents differs by region, with intravenous ajmaline and flecainide being the most commonly utilized (Brugada et al., 2018; Poli et al., 2018). This diagnostic procedure must be carried out with continuous electrocardiographic supervision and is deemed positive if a Type 1 ECG pattern emerges during the administration of the drug. Additionally, the appearance of frequent ventricular premature beats or more intricate ventricular arrhythmias during the test serves as a critical indicator to discontinue the drug to prevent the onset of ventricular arrhythmias (Brugada et al., 2018). It is important to highlight that approximately one-quarter of these pharmacological induction tests may yield false-negative results (Chauveau et al., 2017).

Before the year 2013, the guidelines for diagnosing Brugada Syndrome stipulated that confirmation of the condition required the identification of at least one among six additional clinical factors. These included: (1) documented episodes of ventricular fibrillation or tachycardia, (2) early familial occurrences of sudden cardiac death before the age of 45, (3) the presence of coved-type electrocardiogram patterns within the family, (4) the ability to induce ventricular tachycardia through programmed electrical stimulation tests, (5) instances of syncope, and (6) episodes of nocturnal agonal breathing observed in patients (Mazzanti and Priori, 2016; Korlipara et al., 2021; Vohra et al., 2015). Even though these specific clinical indicators are not part of the current diagnostic framework for BrS, many authorities in the field recommend the inclusion of distinct symptoms to refine diagnostic protocols. Such symptoms include confirmed cases of VT/VF, recorded syncope episodes, cardiac arrests, nocturnal agonal respiration, or a substantiated familial history, thereby potentially enhancing both the precision and dependability of diagnosing BrS (Adler, 2016; Polovina et al., 2017; Korlipara et al., 2021).

**FIGURE 1:**
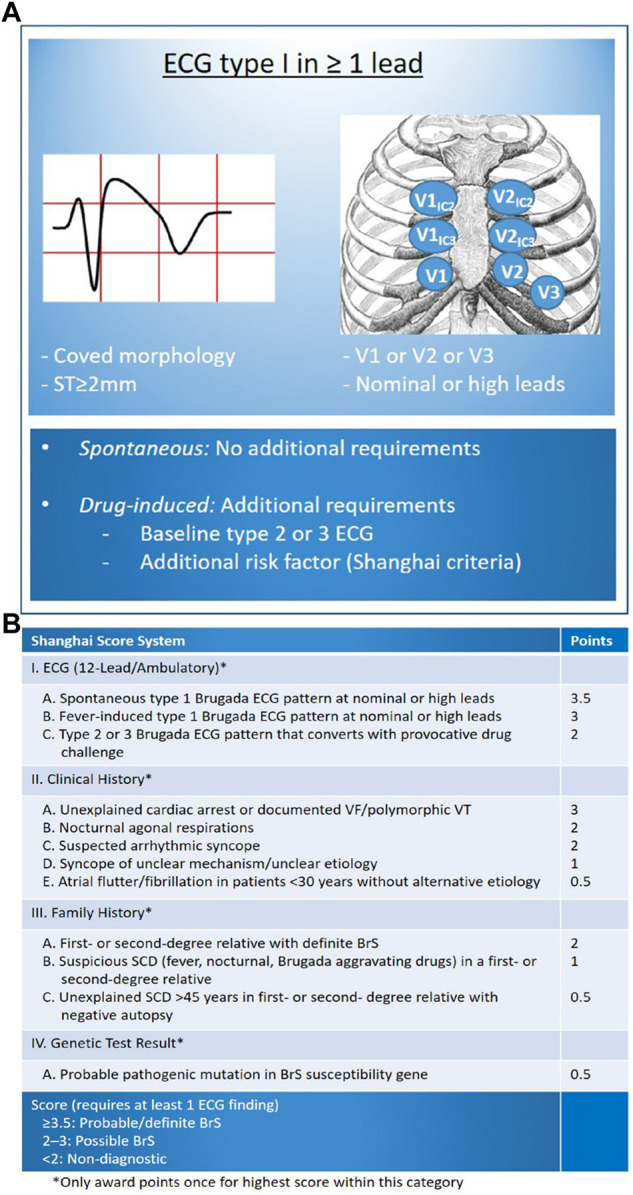
Diagnosis of BrS based on Latest Expert Consensus Report (Antzelevitch et al., 2016b). **(A)** Display standard flow chart; **(B)** Shanghai Score System including other risk factors requiring BrS diagnosis (Marsman et al., 2022). (Reproduced with permission from: E Madelief J Marsman et al. BrS: update and future perspectives. Heart 2022; 108:668–675).

Throughout the years, the conceptual framework and diagnostic standards for BrS have undergone revisions. The latest expert consensus statement outlines the following diagnostic criteria (Figure 1A) (Antzelevitch et al., 2016b): (1) Identification of a spontaneous Type 1 BrS-ECG, characterized by a coved-type pattern, or (2) Detection of a Type 1 BrS-ECG pattern that is revealed through the administration of sodium channel blockers or during febrile episodes. This second criterion is applicable only when the patient initially presents with a Type 2 ECG pattern and is accompanied by at least one additional factor as specified in the “Shanghai Score System” (refer to Figure 1B) (Marsman et al., 2022).

Shanghai Score System was released in 2016 and the scoring system was based on ECG, family history, clinical symptoms and genetics: for probable/definite BrS with the score ≥3.5; for possible BrS with the score of 2∼3; for non-diagnostic BrS with the score <2 (Chatterjee et al., 2020; Kawada et al., 2018; Wilde, 2018). Notably, Shanghai Score System is not universally accepted and a large percentage of medical centers continue to diagnose BrS based only on type 1 ECG (Walia et al., 2019).

## 7 Differential diagnosis

Various acute and chronic medical conditions can produce ECG patterns that resemble those seen in BrS. Among the acute conditions, the most frequently encountered are acute coronary syndromes, pericarditis, myocarditis, pulmonary embolism, and dissecting aortic aneurysm. In these instances, once the underlying acute condition is effectively managed, the ECG abnormalities typically normalize (Cerrone et al., 2022). On the other hand, persistent or long-term conditions that can mimic BrS on an ECG include left ventricular hypertrophy, the physiological adaptations seen in athlete’s heart, right bundle branch block, and certain cardiomyopathies such as arrhythmogenic right ventricular cardiomyopathy (Cerrone et al., 2022; Baranchuk et al., 2012). Furthermore, factors like fever, increased vagal tone, disturbances in electrolyte levels, intoxication with substances like alcohol or cocaine, and the use of specific Class I antiarrhythmic medications (including sodium channel blockers) can reveal or exacerbate BrS-like ECG patterns (Cerrone et al., 2022; Brugada et al., 2018).

## 8 Risk stratification

All BrS patients should receive risk stratification to identify further treatment options. The initial assessment should be based on their medical history. Those BrS patients with a history of cardiac arrest, arrhythmia-induced syncope or ventricular arrhythmia have the highest risk for future VT/VF (Marsman et al., 2022), with the incidence of recurrence in 10 year period as high as 48% (Frédéric et al., 2013). The history of arrhythmic syncope in patients with BrS type 1 ECG pattern has been considered to be a risk factor for SCD (Kabra et al., 2020). Studies have shown that syncope alone increases the risk of ventricular arrhythmias, with an annual incidence of 1.9% (Probst et al., 2010). Vasovagal or nerve-mediated syncope must be ruled out (Brugada et al., 2018). No further stratification is necessary for patients with the above symptoms because they require implantable cardioverter defibrillator (ICD) implantation for secondary prevention (Kabra et al., 2020).

Numerous research studies have indicated that individuals exhibiting a spontaneous Type 1 ECG pattern characteristic of BrS are at a considerably higher risk of experiencing adverse events compared to those who display a Type 1 ECG pattern induced by pharmacological agents (Probst et al., 2010; Delise et al., 2011).

Additional distinct factors that increase the likelihood of adverse outcomes in patients with BrS include the detection of fragmented QRS complexes and early repolarization patterns in inferior or lateral ECG leads. These ECG characteristics are frequently observed in high-risk BrS individuals, with occurrence rates reaching up to 27% (Conte et al., 2016; Rattanawong et al., 2018). Furthermore, a ventricular refractory period shorter than 200 milliseconds, ST segment elevations during the recovery phase of a stress test, prolonged TpTe, and the presence of atrial fibrillation, which is seen in as many as 54% of BrS patients, have also been linked to poorer prognoses in this population (Arbelo and Brugada, 2014; Zumhagen et al., 2016). In a comprehensive cohort study involving 500 BrS patients, Pannone et al. (2024) reported a 20.8% prevalence of SCN5A gene variants. Patients carrying predicted loss-of-function mutations in SCN5A as well as in other genes demonstrated a heightened risk of ventricular arrhythmias. These loss-of-function variants in either SCN5A or non-SCN5A genes independently predicted the occurrence of VA during follow-up. Consequently, individuals with such genetic alterations may necessitate more rigorous monitoring due to their elevated arrhythmic risk and require a meticulous assessment of the arrhythmogenic substrate when being considered for ablation procedures.

In recent developments, Sieira and colleagues (Sieira and Brugada, 2017) introduced a sophisticated risk assessment model designed to improve the stratification and management of patients diagnosed with BrS. This model incorporates six distinct factors that are linked to an increased likelihood of adverse outcomes: (1) episodes of syncope, (2) aborted SCD, (3) presence of a spontaneous Type 1 BrS-ECG pattern, (4) dysfunction of the sinus node (SND), (5) early family history of SCD among first-degree relatives, and (6) inducible ventricular arrhythmias. The model demonstrated a predictive accuracy with an area under the curve (AUC) of 0.82, indicating that individuals scoring above 2 have a 9.2% probability of experiencing adverse events within 5 years. For patients identified as high-risk through this model, implantation of an implantable cardioverter defibrillator (ICD) is considered a prudent therapeutic strategy. Although the Sieira Score is derived from data collected at a single center over nearly 3 decades, which includes consistently structured and repetitive patient treatment protocols, its applicability might differ from multicenter studies that involve diverse methodologies and patient characteristics. The role of electrophysiological studies (EPS) in predicting outcomes has been a subject of ongoing debate (Marsman et al., 2022). According to Sieira et al. (2015), the inability to provoke ventricular arrhythmias during EPS was associated with a 98% negative predictive value over a 5 year follow-up period, yet EPS does not consistently identify patients with low risk (Sroubek et al., 2016). The FINGER Registry, encompassing data from France, Italy, Netherlands, and Germany, did not find that family history of SCD or predictors induced by EPS were significant (Probst et al., 2010). In a comprehensive pooled analysis involving more than 1,300 BrS patients, Sroubek et al. (Sroubek et al., 2016) elaborated that arrhythmias induced during EPS correlated with a two to threefold increase in the risk of sudden cardiac arrest or ICD shocks due to ventricular tachyarrhythmias over a 38 month period. Furthermore, Wu et al. (2016) reported that ventricular arrhythmias triggered by EPS and spontaneous Type 1 BrS-ECG patterns were both associated with a heightened risk of developing future cardiac complications.

## 9 Treatment

It is strongly advised that individuals diagnosed with BrS refrain from exposure to factors that may precipitate VF and SCD (Postema et al., 2009). Current clinical guidelines outline three principal therapeutic approaches for managing BrS patients: (1) implantation of an ICD, (2) radiofrequency ablation procedures, and (3) pharmacological interventions (Korlipara et al., 2021). Furthermore, research conducted by Sara et al. (D’Imperio et al., 2021b) demonstrated that adopting healthy dietary practices, such as moderating food intake, following a ketogenic diet, and limiting alcohol consumption, can contribute to a decreased frequency of arrhythmic events in patients with BrS.

### 9.1 ICD therapy

The 2015 ESC Guidelines provide a structured classification for the use of ICDs in patients diagnosed with BrS. Specifically, ICD implantation is categorized as a Class I recommendation for individuals who have survived an aborted cardiac arrest or who exhibit spontaneously sustained VT (Polovina et al., 2017; Korlipara et al., 2021). For patients who present with a spontaneous Type 1 ECG pattern coupled with a history of syncope, ICD placement is deemed a Class IIa indication. Additionally, ICD implantation is considered a Class IIb recommendation for those who develop VF during PES with the application of two or three extra stimuli at two distinct anatomical sites (Polovina et al., 2017; Korlipara et al., 2021; Pappone and Santinelli, 2017). Conversely, patients identified as low-risk, characterized solely by drug-induced BrS ECG patterns irrespective of their family medical history or specific gene mutations, do not require ICD implantation (Al-Khatib et al., 2017). However, the procedure of ICD implantation is not without its drawbacks, as it is associated with several potential complications. These include inappropriate shocks, device-related issues, and psychological burdens that can adversely affect the patient’s quality of life (Korlipara et al., 2021). In a substantial single-center cohort study focusing on ICD implantation in younger BrS patients, it was found that 20% of the participants experienced inappropriate shocks, and 14% encountered device-related complications over a 7 year follow-up period (Gonzalez Corcia et al., 2017).

### 9.2 Radiofrequency ablation therapy

Over the past 20 years, radiofrequency ablation has become an increasingly recognized treatment modality for BrS. This approach focuses on eliminating focal arrhythmogenic substrates and is particularly beneficial for BrS patients who experience significant symptoms, such as electrical storms or frequent ICD shocks, especially after conventional therapies have failed, thus receiving a Class IIb recommendation (Pappone and Brugada, 2017; Korlipara et al., 2021). A recent study by Nademanee (2021) identified the anterior epicardial region of the RVOT as the primary target for ablation. Targeting this area not only leads to the normalization of ECG readings post-procedure but also significantly reduces the recurrence of VF in BrS patients. It is noteworthy that approximately 35% of BrS individuals harbor arrhythmogenic substrates, which manifest as Abn-Egm patterns in both the body and inferior segments of the RV (Nademanee et al., 2016). Despite these advancements, accurately identifying the lesion sites and ensuring the preservation of healthy myocardial tissue remain ongoing challenges. Furthermore, radiofrequency ablation necessitates long-term follow-up data to fully establish its efficacy and safety profile before it can be widely adopted as a standard alternative to ICD implantation.

### 9.3 Medication

It is mainly used for the following three situations: (1) treatment for acute severe arrhythmia, (2) prevention of arrhythmia events in patients who have been repeatedly shocked by ICD, and (3) use in patients who are contraindicated or refuse to use ICD (Brugada et al., 2018; Belhassen et al., 2004). Quinidine and isoproterenol are endorsed as therapeutic options for patients with BrS exhibiting ventricular dysfunction, according to the 2006 guidelines from the American College of Cardiology, American Heart Association, and European Society of Cardiology (Mashar et al., 2014). These medications are effective in halting electrical storms, which are defined as three or more continuous episodes of VT or VF, or by receiving appropriate ICD shocks within a 24 h timeframe (Shelke et al., 2018). Quinidine, classified as a Class Ia antiarrhythmic agent, operates by blocking ITo and I-Kr channels, thereby preventing the induction of VF and suppressing spontaneous VA in clinical practice (Coppola et al., 2021). Lower doses of quinidine (<600 mg) have been explored as an alternative to mitigate gastrointestinal side effects associated with higher dosages (Márquez et al., 2012). When well-tolerated, the recommended dosage ranges from 600 to 900 mg (Viskin et al., 2009). However, recent research by Mazzanti et al. (2019) demonstrated that even minimal doses of quinidine significantly reduce the recurrence of life-threatening arrhythmic events in patients who have previously experienced such episodes over the long term. Notably, approximately 15% of patients in their study cohort continued to experience life-threatening arrhythmic events, suggesting that quinidine does not replace traditional ICD therapy (Cerrone et al., 2022). Additionally, quinidine is associated with several adverse effects, including thrombocytopenia, severe diarrhea, esophagitis, allergic reactions, exacerbation of SND, and the risk of QT interval prolongation and torsade de pointes (Viskin et al., 2013). Interestingly, recent findings from the International SABRUS Registry indicate that quinidine does not effectively prevent SCD caused by BrS (Milman et al., 2017), thereby casting doubt on its prognostic benefits for BrS patients.

Isoproterenol may help control VF storms in BrS by increasing the L-type Ca^2+^ channel (Shimizu et al., 2000). Current guidelines give a class IIa recommendation for the use of isoproterenol in VF storms (Priori and Blomstrom-Lundqvist, 2015).

## 10 Outlook for the future

With the history of more than 30 years, BrShas attracted a wide range of attention from difficult reports to formal nomenclature, making breakthroughs and innovations not only in pathogenesis, diagnosis, risk stratification and treatment, but also in other fields. At present, there is no specific treatment, but it is very helpful for its prevention and intervention. With the continuous innovation of gene detection technology, the explosive growth of gene data and the continuous innovation of methods such as iPS- CM, there are new opportunities and hopes for studying the pathogenesis of BrS and predicting individual risks. For the treatment of BrS, in addition to the treatment protocol defined in the guidelines, it is very important to tailor the treatment protocol. Since existing treatment methods have their own advantages and disadvantages, it is necessary to objectively evaluate the expected clinical efficacies of these different treatment methods for the reference of making the most appropriate decision.
